# 

**Published:** 1881-05

**Authors:** 


### CONTRIBUTORS.

| | | |
|---|---|---|
| Professor | H. C. Allen, | Ann Arbor. |
| " | T. F. Allen, | New York City. |
| Doctor | Edward Bayard, | " " " |
| " | J. B. Bell, | Boston. |
| " | E. W. Berridge, | London. |
| " | A. C. Cowpertwaite, | Iowa City, Iowa. |
| Doctor | Ad. Fellger, | Phila. |
| " | W. H. Jenny, | Kansas City, Mo. |
| " | John Hall, | Toronto, Canada. |
| " | Ad. Lippe. | Phila. |
| " | C. Lippe, | New York City. |
| " | M. MacFarlan, | Phila. |
| " | Thos. Moore, | " |
| " | C. F. Nichols, | Boston. |
| " | C. Pearson, | Washington. |
| " | S. Swan, | New York City. |
| " | Thomas Skinner, | Liverpool. |
| " | P. P. Wells, | Brooklyn. |
| " | Wm. Wesselhoeft, | Boston. |
| " | Wilson, | London. |
| Professor | T. P. Wilson, | Ann Arbor. |

&c. &c. &c.

Authors of accepted papers are furnished with copies of the number in which their articles appear; application for such copies to be made when sending papers.

A prize of fifty dollars, to be adjudged by three physicians of note, will be awarded to best paper published in this Journal during the year 1881

